# The time course of reading processes in children with and without dyslexia: an ERP study

**DOI:** 10.3389/fnhum.2013.00570

**Published:** 2013-10-07

**Authors:** Sandra Hasko, Katarina Groth, Jennifer Bruder, Jürgen Bartling, Gerd Schulte-Körne

**Affiliations:** Department of Child and Adolescent Psychiatry and Psychotherapy, University Hospital MunichMunich, Germany

**Keywords:** developmental dyslexia, phonological lexical decisions, orthography, phonology, dual route model of reading, N170, N400, LPC

## Abstract

The main diagnostic criterion for developmental dyslexia (DD) in transparent orthographies is a remarkable reading speed deficit, which is often accompanied by spelling difficulties. These deficits have been traced back to both deficits in orthographic and phonological processing. For a better understanding of the reading speed deficit in DD it is necessary to clarify which processing steps are degraded in children with DD during reading. In order to address this question the present study used EEG to investigate three reading related ERPs: the N170, N400 and LPC. Twenty-nine children without DD and 52 children with DD performed a phonological lexical decision (PLD)—task, which tapped both orthographic and phonological processing. Children were presented with words, pseudohomophones, pseudowords and false fonts and had to decide whether the presented stimulus sounded like an existing German word or not. Compared to control children, children with DD showed deficits in all the investigated ERPs. Firstly, a diminished mean area under the curve for the word material-false font contrasts in the time window of the N170 was observed, indicating a reduced degree of print sensitivity; secondly, N400 amplitudes, as suggested to reflect the access to the orthographic lexicon and grapheme-phoneme conversion, were attenuated; and lastly, phonological access as indexed by the LPC was degraded in children with DD. Processing differences dependent on the linguistic material in children without DD were observed only in the LPC, suggesting that similar reading processes were adopted independent of orthographic familiarity. The results of this study suggest that effective treatment should include both orthographic and phonological training. Furthermore, more longitudinal studies utilizing the same task and stimuli are needed to clarify how these processing steps and their time course change during reading development.

## Introduction

Reading and writing are fundamental skills for daily life that allow us to integrate properly into a community and they are crucial for acquiring knowledge and transmitting information. Although reading and spelling require highly complex processes (Massaro and Cohen, 1994), most children acquire these skills without any serious problems. However, despite adequate teaching some children fail to develop age appropriate reading and spelling skills. These children suffer from developmental dyslexia (DD), which is one of the most common specific developmental disorders affecting around 4–9% of school-aged children (Shaywitz et al., 1990; Katusic et al., 2001; Esser et al., 2002). DD is characterized by severe problems in learning to read properly and is often accompanied by a comorbid spelling disorder. These difficulties are not the direct result of below-average general intelligence, inadequate schooling and neurological or sensory deficits (Dilling, 2006). DD accompanies the individuals throughout their lifespan and interferes with academic achievement, professional success and mental health (Esser et al., 2002).

Efforts to pinpoint the underlying mechanisms of DD have resulted in a substantial body of evidence that points toward a phonological core deficit (Snowling, 2001; Ramus et al., 2003; Vellutino et al., 2004). According to the phonological deficit hypothesis it is assumed that subjects with DD have difficulties in applying grapheme-phoneme correspondence rules due to an underspecification of phonological representations, an impaired access to these phonological representations (Ramus and Szenkovits, 2008) or a deficient association of letters and speech sounds (Blau et al., 2010; Froyen et al., 2011; for review see Blomert, 2011).

Orthographic consistency of a language influences the nature of reading difficulties. DD in regular orthographies, such as German, is mainly characterized by a remarkable reading speed deficit or rather an impaired acquisition of automatic reading (Wimmer, 1993, 1996; Landerl et al., 1997; Landerl, 2001; Bergmann and Wimmer, 2008; for review see Wimmer and Schurz, 2010) as well as faulty spelling (Klicpera and Gasteiger-Klicpera, 1998; Schulte-Körne, 2002). Spelling difficulties in transparent orthographies point to an orthographic core deficit (e.g., Bergmann and Wimmer, 2008; Bekebrede et al., 2009; van der Mark et al., 2009). A growing body of evidence suggests that subjects with DD are marked by poorer and less specified orthographic representations and delayed or impaired access to available orthographic representations (Bergmann and Wimmer, 2008; Bekebrede et al., 2009; van der Mark et al., 2009; Marinus and de Jong, 2010).

The phonological lexical decision (PLD)—task seems especially appropriate to investigate orthographic and phonological processing during reading. In the PLD—task, used in the present study, subjects are presented with real words (W), pseudohomophones (PH), pseudowords (PW) and false fonts (FF) and indicate whether the visually presented stimulus sounds like a real word or not (Kronbichler et al., 2007; van der Mark et al., 2009, 2011; Schurz et al., 2010; Wimmer et al., 2010). The PLD—task taps orthographic processing (i.e., the processing of orthographic material) on two levels. Firstly, by comparing the letter string material (W; PH; PW) to the visual control stimuli (FF) print sensitivity will be examined. Secondly, the contrast between orthographic familiar (W) and unfamiliar (PH; PW) word material provides information about the subjects' familiarity with orthographic representations. Furthermore, according to dual route models of reading (e.g., Coltheart et al., 1993, 2001) contrasting of unfamiliar (PH; PW) with familiar (W) word material also taps phonological processing because grapheme-phoneme correspondence rules need to be applied in order to sound out the orthographic unfamiliar word material. Because PH and PW were derived from real W it is possible that they were read by mapping larger units, such as bigrams and trigrams to phonology. However, the reading process remains sublexical.

The PLD—task has been employed in a number of fMRI studies (Kronbichler et al., 2007; Bruno et al., 2008; van der Mark et al., 2009, 2011; Wimmer et al., 2010). In subjects with DD, results point to a reduced print sensitivity as indicated by a lack of higher activity for linguistic material (W; PH; PW) in contrast to FF. And results also indicate an absence of orthographic familiarity as indexed by a lack of decreased activation for orthographic familiar (W) in contrast to orthographic unfamiliar (PH; PW) letter strings in the visual word form area (VWFA; van der Mark et al., 2009; Wimmer et al., 2010). Furthermore, results indicate deficits in phonological processing as suggested by a hemodynamic hypoactivation in response to PH and PW compared to subjects without DD in the left inferior frontal gyrus (Wimmer et al., 2010). On the behavioral level prolonged reaction times for W, PH, and PW were found in subjects with DD (Bergmann and Wimmer, 2008; van der Mark et al., 2009, 2011; Wimmer et al., 2010). Although reaction times were prolonged, the response pattern (W < PH < PW) was similar to control subjects suggesting that subjects with DD relied on comparable reading processes. Thus, these findings seem to highlight an impairment in the speed of access to orthographic and phonological representations in DD (Bergmann and Wimmer, 2008; van der Mark et al., 2009, 2011; Wimmer et al., 2010).

Keeping the reading speed deficit as the main diagnostic criterion for DD in transparent orthographies in mind, it is necessary to understand how the temporal course during reading might differ in DD, thus clarifying whether any steps in the reading process are degraded in children with DD. Identifying impaired processing steps as well as their dependencies during the time course of reading processes is essential for effective intervention as this knowledge might help to derive implications for choosing appropriate treatment methods. Due to the high temporal resolution providing a real-time measure of neural processes event-related-potentials (ERPs) are adapted to disentangle single processing steps. The aim of the present study was to investigate the time course of orthographic and phonological processing in order to provide a temporal model of reading processes in normal developing children and to further identify whether any steps in the reading process are degraded in children with DD. In order to cover different processes which are associated with reading we decided to investigate three reading related ERPs using the PLD—task: the N170, N400, and LPC.

The N170 is the first ERP component thought to reflect orthographic processes (e.g., Bentin et al., 1999; Maurer et al., 2005a,b). It is recorded over left occipito-temporal brain regions and peaks around 170 ms after stimulus onset in skilled adult readers (Bentin et al., 1999; Maurer et al., 2005a,b). The N170 distinguishes letter strings from low-level visual control stimuli (e.g., symbol strings: Tarkiainen et al., 1999; Maurer et al., 2005a,b; forms: Bentin et al., 1999; alphanumeric symbols: Bentin et al., 1999; shapes: Eulitz et al., 2000 and dots: Eulitz et al., 2000). Amplitudes were higher for letter strings, thus implicating that the left lateralized N170 is sensitive to print. Whether the N170 is sensitive to familiar orthographic material is not clear. Some studies described larger amplitudes in response to consonant strings (McCandliss et al., 1997) and pseudowords (Compton et al., 1991) compared to familiar words, as well as larger amplitudes for low frequency words compared to high frequency words (Sereno et al., 1998; Hauk and Pulvermüller, 2004). However, some research did not report amplitude differences between words, pseudowords and consonant strings (Nobre et al., 1994; Salmelin et al., 1996; Bentin et al., 1999; Cornelissen et al., 2003; Maurer et al., 2005b). Varying task requirements might lead to the contrasting results (Maurer and McCandliss, 2008).

The print sensitivity of the N170 develops together with reading acquisition, as children learn to integrate orthographic and phonological information of words. In preschool children N170 amplitudes do not differ between words and symbol strings (Maurer et al., 2005b, 2006). At the end of second grade, however, peak amplitudes are higher for words compared to symbol strings. Furthermore, in contrast to adults where a left lateralization is observed, in children the N170 is symmetrically distributed over occipito-temporal regions (Maurer et al., 2006) with a delay of 50 ms (Maurer et al., 2005b, 2006, 2007; Brem et al., 2009).

N170 amplitudes were found to be reduced in 8-year-old second graders with DD (Maurer et al., 2007), but not in fifth grade children with DD (Maurer et al., 2011; Hasko et al., 2012), suggesting that reduced print sensitivity plays a role especially in the early stage of reading acquisition and neurophysiological deficits related to DD change during development (Maurer et al., 2011). These results point to a delayed specialization for processing letter strings in DD. However, there is also evidence that print sensitivity is still reduced in pre-adolescents (age 9–13, mean age 10.7; Araújo et al., 2012) and adults (Helenius et al., 1999; Mahé et al., 2012) with DD, thus contradicting the hypothesis of a delayed specialization for processing letter strings in DD. Interestingly studies reporting on N170 impairments in adults with DD (Helenius et al., 1999; Mahé et al., 2012) included subjects with more severe reading deficits (at least two standard deviations below the mean) compared to studies which did not report on N170 impairments (Maurer et al., 2011; Hasko et al., 2012). This suggests that the N170 impairment might be also influenced by the degree of reading and spelling impairments (Mahé et al., 2012).

The N400 is recorded over centro-parietal electrodes during written and spoken language processing (Deacon et al., 2004; for review see Lau et al., 2008; Kutas and Federmeier, 2011). This component was investigated in a large number of studies employing different tasks. It was found to be elicited by semantic incongruity (e.g., Kutas and Hillyard, 1980; Brandeis et al., 1994; Schulz et al., 2008), orthographic and phonological manipulations (e.g., Rugg and Barrett, 1987; Praamstra and Stegeman, 1993; Dumay et al., 2001; Bonte and Blomert, 2004; Rüsseler et al., 2007) as well as by orthographically and phonologically legal pseudowords, which do not possess an entry in the mental lexicon (Holcomb and Neville, 1990; Doyle et al., 1996; Deacon et al., 2004; for review see Kutas and Federmeier, 2011). As being sensitive to all of these properties it is still unclear whether the N400 might reflect lexical or post-lexical processing or even both. N400 effects have been reported in children as young as 12 months (Friedrich and Friederici, 2010) and N400 amplitudes and latencies decrease across development (Holcomb et al., 1992; Juottonen et al., 1996; Hahne et al., 2004; Atchley et al., 2006). In visual lexical decision tasks N400 amplitudes were found to be smaller to orthographic familiar compared to orthographic unfamiliar word forms in adults (e.g., Braun et al., 2006; Briesemeister et al., 2009). Therefore, it could be interpreted that the N400 amplitudes elicited in visual lexical decision tasks reflect lexical processing, rather than post-lexical processing, because in the latter case one would have expected comparable N400 amplitudes for W and PH, which share phonology and meaning. In 7-year-old children the N400 amplitude was not modulated by orthographic familiarity (Coch and Holcomb, 2003).

With respect to DD results regarding N400 effects are rather inconsistent. In a variety of studies reduced N400 amplitudes are reported across different language tasks in children (visual rhyme matching task: Ackerman et al., 1994; reading of correct and incorrect sentence endings: Brandeis et al., 1994; Schulz et al., 2008) and adults with DD (visual semantic, rhyme, and definite article judgment task: Rüsseler et al., 2007; visual word recognition task: Johannes et al., 1995) in contrast to control subjects. Other authors, however, did not confirm the abnormal N400 activation in children (listening to sentences with semantic violation: Sabisch et al., 2006; word categorization task: Silva-Pereyra et al., 2003; auditory lexical decision task: Bonte and Blomert, 2004) and adults with DD (word recognition task: Rüsseler et al., 2003). Neville et al. (1993) found even higher N400 amplitudes during reading incongruent sentence endings in 8- to 10-year-old children with DD and language impairments, suggesting maturational changes during development influencing the N400. Study inconsistencies in N400 response could be contributed to a number of factors including task and stimulus type, presentation modality, severity of reading impairment and age.

The N400 is followed by a late positive complex (LPC), which occurs in a time window between 500 and 800 ms and is distributed over the left centro-parietal scalp in adults (Friedman and Johnson, 2000; Finnigan et al., 2002; Rüsseler et al., 2003; Yonelinas et al., 2005; Balass et al., 2010; for review see: Rugg and Curran, 2007), adolescents (Schulte-Körne et al., 2004) and children (Hepworth et al., 2001; van Strien et al., 2009). The LPC might be involved in word recognition memory as LPC amplitudes are higher to correctly recognized old words compared to new words (for review see Rugg and Curran, 2007). This effect is not dependent on intentional retrieval (Curran, 1999).

LPC amplitudes were reduced in adolescents (Schulte-Körne et al., 2004) and adults with DD (Rüsseler et al., 2003) or low reading skills (Perfetti et al., 2005; Balass et al., 2010). For example, Schulte-Körne et al. (2004) investigated tenth graders with and without a history of DD. In a learning phase participants had to study a list of simple pseudowords and graphic symbols. In the recognition phase the learned items were presented together with new items and participants decided whether the presented item was new or learned. Interestingly, all subjects performed the task equally well, however, the LPC was attenuated in response to learned pseudowords in adolescents with DD compared to adolescents without DD. No group differences were found for graphic symbols. These results were interpreted as reflecting a specific word recognition memory deficit (Schulte-Körne et al., 2004). In the present study we did not investigate a word recognition task but a PLD—task, thus the LPC elicited in the present study might reflect the access to the phonological lexicon and the recognition of a phonological entry of an existing German word.

Taken together a large body of evidence points to deficits in different processing steps during reading in subjects with DD. As reviewed these studies often focused their investigation on one single process or used different tasks in order to explore different processing steps, thus also leading to inconsistent results. To the best of our knowledge this is the first study investigating the PLD—task in children without and with DD using ERPs. One major advantage of the PLD—task, is the fact, that it is a continuous reading task, which allows to study both orthographic and phonological processing in one experiment, thus avoiding confounding effects due to varying attention, motivation or arousal levels or due to different task demands and stimuli properties.

We predicted to find processing differences between the stimuli and groups on both the neurophysiological and the behavioral level. On the neurophysiological level we expected to find higher amplitudes for letter strings (W; PH; PW) compared to FF in the time window of the N170 over occipito-temporal electrodes in children without DD, as an index of print sensitivity. If the N170 is also sensitive to orthographic familiarity in children we hypothesized to find decreased amplitudes for orthographic familiar (W) in contrast to orthographic unfamiliar (PH; PW) word material. For children with DD we expect to find no print sensitivity and orthographic familiarity effect on the N170 component. Furthermore, we expected to find an N400 over centro-parietal electrodes in normal developing children reflecting lexical or post-lexical processing. If the N400 indexes lexical processing stages, we expected to find lower amplitudes for orthographic familiar (W) compared to orthographic unfamiliar (PH; PW) word material. If the N400 indicates post-lexical processing we hypothesized to find amplitude differences between phonological familiar (W; PH) and phonological unfamiliar word material (PW). Findings whether the processing steps related to the N400 are deficient in children with DD are inconsistent. If processing steps related to N400 are degraded in children with DD, we would expect them to show attenuated N400 amplitudes compared to normal developing children. Finally, we hypothesized to find higher LPC amplitudes over left centro-parietal electrodes for W and PH in control children, indicating successful access to the phonological lexicon. However, this pattern of activation is not expected for the children with DD. Against this background we anticipated delayed reaction times and reduced accuracy rates for W, PH, and PW in children with DD in contrast to control subjects. Further, we expected to replicate the reaction time pattern observed in former studies (W < PH < PW).

## Materials and methods

### Participants

As part of a longitudinal study of our research group (see Groth et al., 2013) contact details of all children born in Munich between January 2000 and December 2003 were requested from the Department of Public Order of Munich. Approximately 10,000 randomly selected families were contacted via letter and asked for participation in the present study. Additionally, study information was sent to schools, pediatrics, child psychiatrists and psychologists and socio-pediatric facilities.

Recruitment procedure had two stages. In a first step, families who expressed their interest in the present study underwent a telephone interview. Potential participants were excluded from the next stage of recruitment if one of the parents indicated that his or her child had a history of specific language disorder, had been treated for any neurological or psychiatric disorder or was currently under medication. To ensure that the children did not suffer from symptoms of Attention-Deficit Hyperactivity Disorder (ADHD) the parents were asked to estimate their children on the subscale “Attention Problems” of the Child-Behavior-Checklist (CBCL/1–4; Achenbach, 1991). Children were excluded if they scored above average in the parent questionnaire (CBCL-score >7 for girls and CBCL-score >8 for boys) indicating a risk of ADHD. Furthermore, participants had to be German native speakers, had to attend the second grade, their hearing had to be normal and their vision had to be normal or corrected-to-normal. We decided to recruit children at the end of second grade, because at this point in time there is a high level of certainty regarding the stability of the DD diagnosis.

In the second recruitment step 250 second graders were invited and screened regarding their reading and spelling performance as well as their non-verbal intelligence. Inclusion criteria for all children were an IQ score within the normal range (≥85 IQ points) as measured with the Culture Fair Intelligence Test (CFT 1; Cattell et al., 1997). Furthermore, common word reading fluency and spelling were used as inclusion criteria. Common word and pseudoword reading fluency was assessed by using a German standardized one-minute-fluent reading-test (German: Ein-Minuten-Leseflüssigkeitstest [SLRT-II]; Moll and Landerl, 2010). In this measure, children are presented with a list of common words and pseudowords and are given one minute to read as many items as possible. Spelling was assessed with a German standardized basic vocabulary spelling test for grades 2–3 (German: Weingartener Grundwortschatz Rechtschreib-Test für zweite und dritte Klassen [WRT2+]; Birkel, 1994). In addition, reading comprehension measured with a German standardized reading comprehension test for grades 1–6 (German: Ein Leseverständnistest für Erst- bis Sechstklässler [ELFE 1–6]; Lenhard and Schneider, 2006) was assessed.

In order to ensure inclusion of only truly average (or above average) readers and spellers in our control sample, children belonging to the control group were required to be within 0.70 standard deviations of the lower end of the norm scale calculated in *T*-values (mean = 50; *SD* = 10; cutoff criteria was therefore set to a *T*-value of 43). In order to be included in the group of children with DD, participants had to fulfill the diagnosis of DD according to the International Classification of Diseases (ICD-10: F 81.0; Dilling, 2006). Their reading and spelling score had to diverge from the mean *T*-value for at least one standard deviation (1 SD; cutoff criteria was therefore set to a *T*-value of 40) and 1 SD from the IQ according to the regression criterion (Schulte-Körne et al., 2001). Thus, both a discrepancy of reading and spelling abilities from the class or age level, but also from the level expected on the basis of the child's intelligence is required for diagnosing DD. As the correlation of reading and spelling performance with IQ is not 1, but medium-high the use of a simple discrepancy criterion distorts the diagnostic results for children with low or high intelligence (Schulte-Körne et al., 2001). The application of the regression criterion avoids distortions in extreme ranges by considering the correlation between IQ and reading and spelling abilities. Thus, a higher discrepancy is necessary for children with high intelligence and a lower discrepancy is necessary for children with low intelligence in order to meet the diagnostic criterion of DD (Schulte-Körne et al., 2001). Overall 29 children were included in the control group and 58 children were included in the group of DD. The sample of children with DD was larger compared to the sample of control children because as mentioned above children were recruited as part of a longitudinal study. For the purpose of this longitudinal study children with DD were assigned to three groups. One group received an intensive reading training, a second group performed an intensive spelling training and the third group acted as a control wait-group and received training only after a six month wait period (see Groth et al., 2013 for more information). Here the results of the first point in time, prior to the intervention, will be reported. We therefore decided to compare the control children to the whole group of children with DD. A total of six children from the DD sample were excluded from further analyses due to excessive EEG artifacts, resulting in a sample size of 52 children with DD. All data reported exclude these participants.

Both groups had an average age of about eight years (control group: *M* = 8.15, *SD* = 0.27; group with DD: *M* = 8.30, *SD* = 0.37) and an IQ-score within the normal range. The IQ of control children was significantly higher compared to the IQ of children with DD (see Table 1). In order to control for a confounding influence of the IQ on the ERP results the groups were matched according to their IQ. The Analyses of Variance (ANOVAs) presented below were also run with IQ matched groups and did reveal the same pattern of results. Gender was distributed similarly in both groups (control group: 13 females; group with DD: 21 females). In all reading and spelling tests children with DD performed significantly worse than control children (see Table 1). Apart from one control child and one child with DD all subjects were right-handed.

**Table 1:** **Descriptive statistics of control children and children with DD**.

	**CON (*n* = 29)**		**DD (*n* = 52)**		*p*-value^*^
	***M***	***SD***	***M***	***SD***	
IQ^a^	111.79	10.42	105.35	8.20	=0.003
word reading^b^	56.21	6.76	32.36	3.96	<0.001
pseudoword reading^b^	54.62	7.82	36.33	4.41	<0.001
reading comprehension^c^	56.96	8.03	36.09	4.15	<0.001
spelling^d^	52.04	5.38	34.75	3.94	<0.001

CON, control group; DD, group with DD; n, sample size; M, mean; SD, standard deviation.

a CFT 1.

b SLRT-II.

c ELFE 1–6.

d WRT 2+.

Average reading and spelling scores are delineated by T-values; T-values have a mean of 50 (SD ± 10).

* t-test for independent samples.

Parents and children were informed about the aim, purpose and procedure of the study and gave their written consent prior to inclusion in the study. Children received a present as acknowledgement for their participation. Experimental procedures were approved by the Ethical Committee of the Faculty of Medicine at the University of Munich, Germany.

### ERP paradigm and procedure

**Figure 1:**
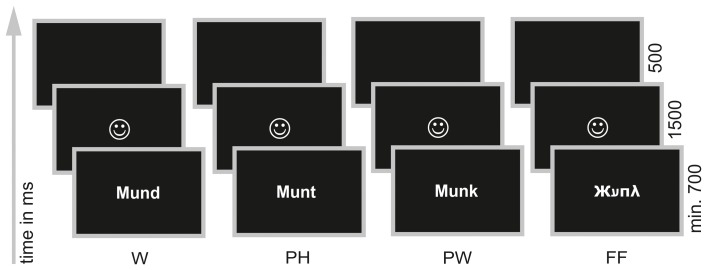
**Phonological lexical decision task.** Words (W; e.g., Mund /m ℧nt/, engl.: mouth), pseudohomophones (PH; e.g., Munt /m ℧nt/), pseudowords (PW; e.g., Munk /m℧ηk/) and false fonts (FF; e.g., 
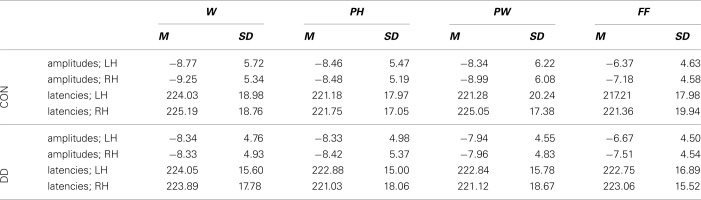
) were presented individually in white on black background in the center of a 17′ screen. Participants were instructed to decide via button press whether a presented stimulus sounded like a real word or not.

During ERP acquisition children performed a PLD—task (Kronbichler et al., 2007; Bergmann and Wimmer, 2008; van der Mark et al., 2009, 2011). In this task participants had to decide whether a visually presented stimulus sounded like a real word or not (“Does … sound like a real word?”, see Figure 1). Children were presented either with words (W; orthographically and phonologically familiar forms of German nouns), pseudohomophones (PH; phonologically correct but orthographically unfamiliar forms of the same words) or pseudowords (PW; phonologically and orthographically unfamiliar forms). W and PH required a “yes” response and PW should be responded with “no.” For each item type (W; PH; PW) 60 stimuli were taken with minor adaptions from the letter strings used in the study of Bergmann and Wimmer (2008) and van der Mark et al. (2009, 2011). Every item was presented once only. In order to avoid a response bias toward “yes” responses we included a fourth condition, consisting of 60 false fonts (FF; van der Mark et al., 2009, 2011) and requiring a “no” response. FF were created by assigning a FF to each upper and lower case letter (van der Mark et al., 2009, 2011, see Appendix for a complete list of all stimuli used in the PLD—task). Furthermore, FF also served as non-lexical control stimuli in order to examine the print sensitivity of the N170 (see Introduction).

According to the “corpus-based word basic form list” (Korpusbasierte Wortgrundformenliste; DeReWo, 2013) compiled on the base of the Mannheim German Reference Corpus (Das Deutsche Referenzkorpus; Kupietz and Keibel, 2009; DeReKo, 2012) nouns used in the present study had a high frequency range, i.e., frequency classes 8–16 (Keibel, 2008). Item length and bigram frequency have a confounding effect on the ERPs of cognitive processes (Johannes et al., 1995; Assadollahi and Pulvermüller, 2003; Hauk and Pulvermüller, 2004; Penolazzi et al., 2007; Proverbio et al., 2008). To avoid effects due to item length and complexity all stimuli were matched for number of characters (3–7 characters). In addition W, PH, and PW were controlled for bigram frequency. Bigram frequencies were also determined based on the Mannheim German Reference Corpus. As can be seen in Table 2 number of characters for all conditions and bigram frequencies for the letter string conditions were equally distributed.

**Table 2:** **Item characteristics for each condition**.

	***W***		***PH***		***PW***		***FF***	
	***M***	***SD***	***M***	***SD***	***M***	***SD***	***M***	***SD***
characters	4.42	0.83	4.43	0.85	4.42	0.83	4.42	0.83
bigram frequency	4.19	2.31	4.45	2.54	4.49	2.59	–	–

W, words; PH, pseudohomophones; PW, pseudowords; FF, false fonts.

All stimuli were presented in white font on black background in the center of a 17′ screen using E-Prime® 2.0 software (Psychology Software Tools, Inc.). The computer screen was placed 70 cm in front of the children resulting in a vertical visual angle of 1.23° and in an average horizontal angle of 3.44°.

The 240 stimuli were presented in two pseudorandomized lists. The order of W and corresponding PH was counterbalanced. In List 1 the W was presented before the corresponding PH in half of the cases and the opposite for the other half. In List 2 the order was reversed (Bergmann and Wimmer, 2008). In addition, a W and its corresponding PH did not appear in close proximity (Bergmann and Wimmer, 2008) and no more than three trials requiring the same response were presented in succession. Half of the children performed List 1, whereas the other half was presented with List 2. Both lists were divided into four blocks, each with 60 stimuli. After each block there was a short break. To ensure that the subjects fully understood the task, the experiment was preceded by a short practice-block (24 trials). Trials utilized in the practice-block did not occur in the experiment.

To make sure that even the poorest reader had enough time to read the letter string stimuli the task was self-paced. However, all children were presented with the stimuli for a minimum of 700 ms in order to guarantee that all participants saw the same in the first milliseconds, which is important for ERP analysis. Participants had to decide by button press whether the presented stimulus sounded like a real word or not. Half of the children used their right hand for giving a “yes” response and the left hand for giving a “no” response, the other half used the left hand for “yes” and the right hand for “no” responses. Depending on correct or incorrect response children were provided with a feedback in form of a happy or sad face (1500 ms). The next trial appeared automatically after a blank screen of 500 ms (see Figure 1).

### ERP recording and analysis

**Figure 2:**
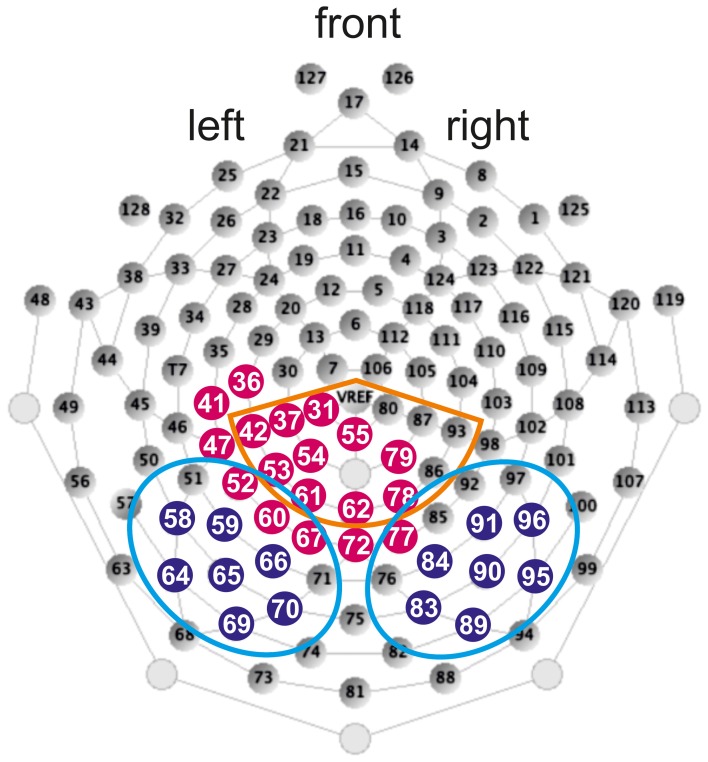
**Illustration of the 128-channel-system and electrode position taken from Electrical Geodesics Inc. (2007).** Filled blue dots depict electrodes included in the LH and RH ROIs of the N170. Light blue circles depict electrodes included in the LH and RH ROIs of the N170 difference waves. Orange circle depicts electrodes included in the ROI of the N400 and filled red dots depict electrodes included in the ROI of the LPC.

EEG was recorded during the stimulus presentation with an Electrical Geodesic Inc. 128-channel-system (see Figure 2 for a schematic illustration of the electrode net). The impedance was kept below 50 kΩ. EEG-data was recorded continuously with Cz as the reference electrode and sampled at 500 Hz. Further analysis steps were performed with Brainvision Analyzer (Brain Products GmbH).

After filtering (low cutoff: 0.5 Hz, time constant 0.3, 12dB/ octave; high cutoff: 40 Hz, 24 dB/ octave; notch filter: 50 Hz; filtered continuous on raw data to avoid discontinuities and transient phenomena), removing EOG-artifacts with Independent Component Analysis (Zhou et al., 2005; Hoffmann and Falkenstein, 2008) and exclusion of other artifacts (gradient criteria: more than 50 μ V difference between two successive data points or more than 150 μ V in a 200 ms window; absolute amplitude criterion: more than ±150 μ V; low activity: less than 0.5 μ V in a 100 ms window), the EEG was re-referenced to the average reference.

The data was then segmented into 1100 ms epochs including 100 ms pre-stimulus baseline and the ERP data was baseline corrected. For inclusion in the statistical analysis a minimum of 20 artifact free trials was necessary. Only correct trials were analyzed. The averages (*M* [*SD*]) for the accepted trials for control children were: W 53.79 [3.10], PH 50.45 [3.74], PW 51.86 [5.03] and FF 56.45 [2.34]. For children with DD an average of 47.23 [4.61], 43.56 [6.64], 40.50 [9.20] and 56.00 [2.31] trials were obtained for the W, PH, PW and FF, respectively. Individual ERPs were averaged per condition (W; PH; PW; FF). Grand averages of all four conditions were computed by averaging separately for each subject group (control group; group with DD).

Based on the electrophysiological activity to W for control children time windows and regions of interest (ROIs) for the N170, N400 and the LPC were determined using running *t*-tests against zero (*p* < 0.05) at each electrode. According to this analysis the time window was set 170–290 ms for the N170, 330–460 ms for the N400 and 600–900 for the LPC. These time windows were applied to all conditions and both groups.

In line with previous studies (e.g., Maurer et al., 2006; Kast et al., 2010; Hasko et al., 2012) the most significant activation of the N170 in the present study was also found bilaterally over occipito-temporal electrodes using the running *t*-tests against zero (*p* < 0.05) for W in control children. According to this activation we defined left and right hemispheric ROIs (LH and RH ROIs). The LH ROI included electrodes 58, 59, 64, 65, 66, 69, 70 and the RH ROI included electrodes 83, 84, 89, 90, 91, 95, 96 (see Figure 2 for exact electrode positions over occipito-temporal sites).

In order to examine the degree of N170 print sensitivity additionally difference waves were calculated between the linguistic material and the non-lexical control stimuli FF for the time window of the N170. ERP difference waves were calculated by subtracting FF from the linguistic material (i.e., W minus FF, PH minus FF, PW minus FF). Furthermore, in order to examine the degree of orthographic familiarity difference waves were calculated between the orthographic familiar (W) and orthographic unfamiliar material (PH; PW). ERP difference waves were calculated by subtracting orthographic unfamiliar material from orthographic familiar material (i.e., W minus PH, W minus PW). Even though both PH and PW are orthographically unfamiliar, difference waves contrasting W and PW might be confounded with phonological and semantic processes, because W and PW do not differ only with respect to orthographic familiarity but also with respect to phonology and semantic. Difference waves were calculated for each child separately and grand averages of all five difference waves (W minus FF, PH minus FF, PW minus FF, W minus PH, W minus PW) were computed by averaging separately for each group (control group; group with DD). Based on the electrophysiological activity to the W minus FF contrast for control children ROIs were determined using running *t*-tests against zero (*p* < 0.05) at each electrode. According to this activation electrodes 51, 52, 58, 59, 60, 64, 65, 66, 67, 69, 70, 71 were comprised in the LH ROI and electrodes 76, 77, 83, 84, 85, 89, 90, 91, 92, 95, 96, 97 were included in the RH ROI (see Figure 2 for exact electrode positions over occipito-temporal sites).

For the N400, according to the running *t*-tests against zero (*p* < 0.05) for W in control children we determined a centro-parietal distribution (see Figure 2; electrodes included in the ROI: 31, 37, 42, 53, 54, 55, 61, 62, 78, 79, 80, 86, 87, 93, 129 (VREF); e.g., Deacon et al., 2004; for review see Lau et al., 2008; Kutas and Federmeier, 2011).

According to the running *t*-tests against zero (*p* < 0.05) for W in control children a left centro-parietal ROI was defined for the LPC (see Figure 2; electrodes included in the ROI: 31, 36, 37, 41, 42, 47, 52, 53, 54, 55, 60, 61, 62, 67, 72, 77, 78, 79; Friedman and Johnson, 2000; Hepworth et al., 2001; Finnigan et al., 2002; Rüsseler et al., 2003; Schulte-Körne et al., 2004; Yonelinas et al., 2005; van Strien et al., 2009; Balass et al., 2010; for review see: Rugg and Curran, 2007).

Mean peak amplitude measures capturing data 20 ms before and 20 ms after the individual peak and latencies were exported for each electrode of the N170 and N400 ROI using the defined time windows. As no clear peak could be observed on the N170 difference waves and on the LPC, we decided to export the area under the curve for each electrode included in the ROI of the N170 difference waves and of the LPC using the defined time windows. The values of individual mean peak amplitudes, latencies, and areas under the curve were averaged after peak export for every ROI.

### Statistical analysis

To test for group differences regarding the N170 mean peak amplitudes and latencies we computed ANOVAs for repeated measures. The ANOVAs included the within-subject factor *condition* (W; PH; PW; FF) and *hemisphere* (LH; RH) and the between-subject factor *group* (control group; group with DD). Similar ANOVAs for repeated measures were run for the mean area under the curve for the N170 difference waves in order to examine the degree of print sensitivity and the degree of orthographic familiarity. For examining the degree of print sensitivity the ANOVA included the within-subject factor *condition* (W minus FF; PH minus FF; PW minus FF) and *hemisphere* (LH; RH) and the between-subject factor *group* (control group; group with DD). For examining the degree of orthographic familiarity the ANOVA included the within-subject factor *condition* (W minus PH; W minus PW) and *hemisphere* (LH; RH) and the between-subject factor *group* (control group; group with DD). N400 mean peak amplitudes and latencies and LPC mean area under the curve were investigated for group differences using ANOVAs for repeated measures. These ANOVAs included the within-subject factor *condition* (W; PH; PW) and the between-subject factor *group* (control group; group with DD). *Post-hoc* analyses were performed with *t*-tests for independent and dependent samples.

The behavioral data (reaction times and accuracy on the PLD—task) was analyzed using ANOVAs for repeated measures including the within-subject factor *condition* (W; PH; PW; FF) and the between-subject factor *group* (control group; group with DD). Trials were excluded from analysis if the response times were lower than 200 ms and deviating more than 2.5 SD from the individual group mean within a condition type. This procedure resulted in a loss of 2.76% of the trials. Furthermore for the reaction time analysis only correct trials were included.

If sample sizes are equal, ANOVAs are unsusceptible against violations of homogeneity of variances. Given that the sample of children with DD was almost twice as big as the control sample the *F*_max_-test was applied in case of violations of the homogeneity of variances (Bühner and Ziegler, 2009). According to the *F*_max_-test an adjustment of the alpha-level is necessary if the critical value of *F*_max_ > 10 is exceeded (Bühner and Ziegler, 2009). In none of the variables the critical value was exceeded. If necessary the Greenhouse-Geisser correction was applied to correct for violations of the sphericity assumption. The alpha level for all analyses was 0.05. In order to avoid alpha-error-inflation due to multiple comparisons the alpha level was corrected using the Bonferroni-Holm correction (Bühner and Ziegler, 2009). In addition to the *p*-values, effect sizes η_p_^2^ for ANOVAs with repeated measures and Cohen's d for *post-hoc t*-tests are reported for significant results (Cohen, 1988; Bühner and Ziegler, 2009).

Furthermore, partial correlations were computed controlling for the factor *group* between the ERP data (N170 mean area under the curve for difference waves; N400 mean peak amplitudes; LPC mean area under the curve) and the behavioral data (common word and pseudoword reading fluency; reading comprehension; spelling). As we did not observe differences between W, PH and PW in the N170 difference waves and in the N400 we decided to use mean values calculated across the three letter string types for the partial correlation analysis. The correlational analysis was exploratory, therefore Bonferroni-Holm correction was not applied. Only significant results (*p* < 0.05) will be reported.

## Results

### ERP data of the PLD—task

#### N170

**Figure 3:**
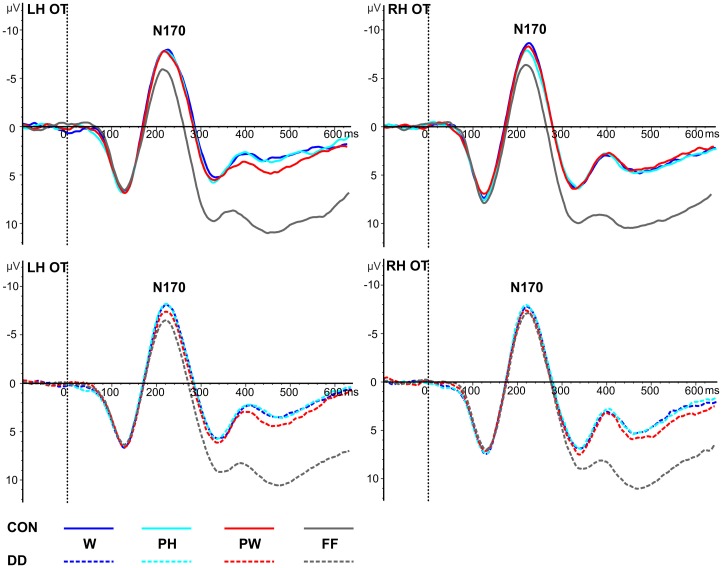
**Illustration of the averages across occipito-temporal (OT) electrodes included in the left hemispheric (LH) and right hemispheric (RH) ROIs of the N170 for control children (CON) and children with DD (DD).** W, words; PH, pseudohomophones; PW, pseudowords; FF, false fonts. Negativity is depicted upwards.

***Mean peak amplitudes.*** In both groups N170 mean peak amplitudes were enhanced for the linguistic material compared to FF (main effect *condition, F*_(3, 237)_ = 15.27, *p* < 0.001, η_*p*_^2^ = 0.16; dependent *post-hoc t*-tests across both groups: FF vs. PW, *t*_(80)_ = 4.14, *p* < 0.001, *d* = 0.46; FF vs. PH, *t*_(80)_ = 5.21, *p* < 0.001, *d* = 0.58; FF vs. W, *t*_(80)_ = 5.59, *p* < 0.001, *d* = 0.63; PW vs. PH, *t*_(80)_ = 0.71, *p* = 0.48; PW vs. W, *t*_(80)_ = 1.48, *p* = 0.14; PH vs. W, *t*_(80)_ = 0.72, *p* = 0.47; see Table 3 and Figure 3). N170 mean peak amplitudes were comparable between *groups*, *F*_(1, 79)_ = 0.08, *p* = 0.78, and distributed symmetrically across both *hemispheres*, *F*_(1, 79)_ = 0.94, *p* = 0.34. No significant interaction between *group* and *condition, F*_(3, 237)_ = 1.50, *p* = 0.22, or *group* and *hemisphere* could be observed, *F*_(1, 79)_ = 0.12, *p* = 0.74.

**Table 3:**
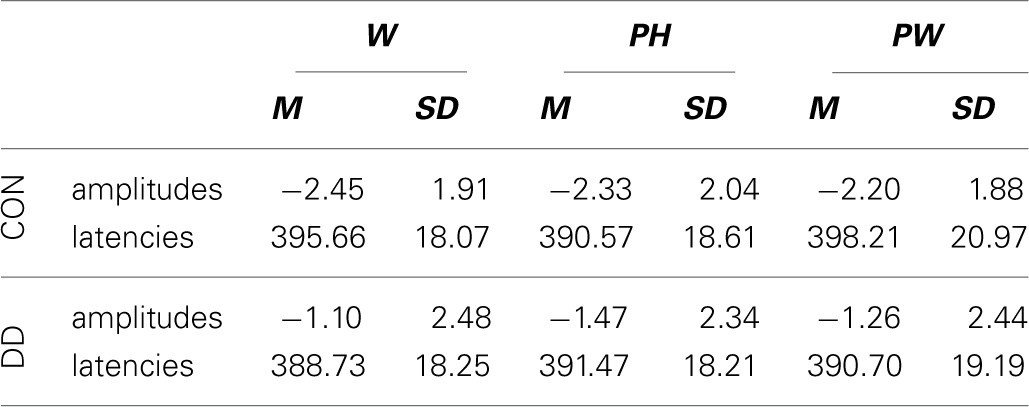
**N170 mean peak amplitudes (μV) and latencies (ms)**.

W, words, PH, pseudohomophones, PW, pseudowords, FF, false fonts; CON, control children, DD, children with DD.

***Peak latencies.*** A significant main effect *condition* occurred, *F*_(2.59, 204.84)_ = 3.65, *p* = 0.018, η _*p*_^2^ = 0.04. Dependent *post-hoc t*-tests revealed signficantly shorter peak latencies only for PH compared to W (FF vs. PW, *t*_(80)_ = −0.64, *p* = 0.53; FF vs. PH, *t*_(80)_ = −0.18, *p* = 0.86; FF vs. W, *t*_(80)_ = −2.62, *p* = 0.01; PW vs. PH, *t*_(80)_ = 0.64, *p* = 0.53; PW vs. W, *t*_(80)_ = −1.98, *p* = 0.05; PH vs. W, *t*_(80)_ = −2.89, *p* = 0.005, *d* = 0.32; see Table 3 and Figure 3). N170 peak latencies were comparable between *groups*, *F*_(1, 79)_ = 0.03, *p* = 0.87, and equal across both *hemispheres*, *F*_(1, 79)_ = 0.32, *p* = 0.57. No significant interaction between *group* and *condition*, *F*_(2.59, 204.84)_ = 2.19, *p* = 0.10, or *group* and *hemisphere*, *F*_(1, 79)_ = 1.43, *p* = 0.24, could be observed.

**Figure 4:**
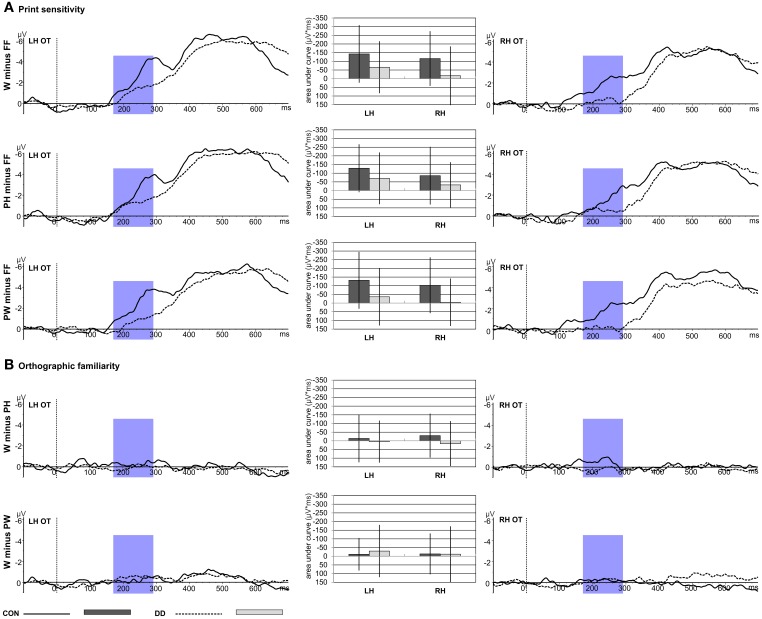
**Print sensitivity and orthographic familiarity for control children (CON) and children with DD (DD).** The time window selected for the N170 difference waves is highlighted in blue (170–290 ms). W, words; PH, pseudohomophones; PW, pseudowords; FF, false fonts; OT, average across occipito-temporal electrodes included in the left hemispheric (LH) and right hemispheric (RH) ROIs of the N170 difference waves. Negativity is depicted upwards. Error bars illustrate standard deviations. **(A)** Depicts the effect of print sensitivity. Difference waves and mean area under the curve are shown for the contrast between the linguistic material and the visual control stimuli (W minus FF; PH minus FF; PW minus FF). **(B)** Depicts the effect of orthographic familiarity. Difference waves and mean area under the curve are shown for the contrast between orthographic familiar and unfamiliar word material (W minus PH; W minus PW).

***Print sensitivity; area under the curve.*** Mean area under the curve was greater for the control group compared to the group with DD for all difference waves contrasting the linguistic material with FF (W minus FF; PH minus FF; PW minus FF; main effect *group, F*_(1, 79)_ = 9.36, *p* = 0.003, η _p_^2^ = 0.11; see Figure 4A). Furthermore, the activation was greater over the left hemisphere compared to the right hemisphere (main effect *hemisphere, F*_(1, 79)_ = 5.08, *p* = 0.027, η _*p*_^2^ = 0.06; see Figure 4A). Mean area under the curve was similar high for all three difference waves, *F*_(2, 158)_ = 0.77, *p* = 0.46. No significant interaction between *group* and *condition*, *F*_(2, 158)_ = 1.27, *p* = 0.28, or *group* and *hemisphere*, *F*_(1, 79)_ = 0.04, *p* = 0.84, could be observed.

***Orthographic familiarity; area under the curve.*** Mean area under the curve was comparable high for both *groups*, *F*_(1, 79)_ = 0.29, *p* = 0.59, and *hemispheres*, *F*_(1, 79)_ = 0.03, *p* = 0.85. Furthermore, mean area under the curve was similar for W minus PH and W minus PW, *F*_(1, 79)_ = 0.56, *p* = 0.46 (see Figure 4B). No significant interaction between *group* and *condition*, *F*_(1, 79)_ = 2.05, *p* = 0.16, or *group* and *hemisphere*, *F*_(1, 79)_ = 0.66, *p* = 0.42, could be observed.

#### N400

**Figure 5:**
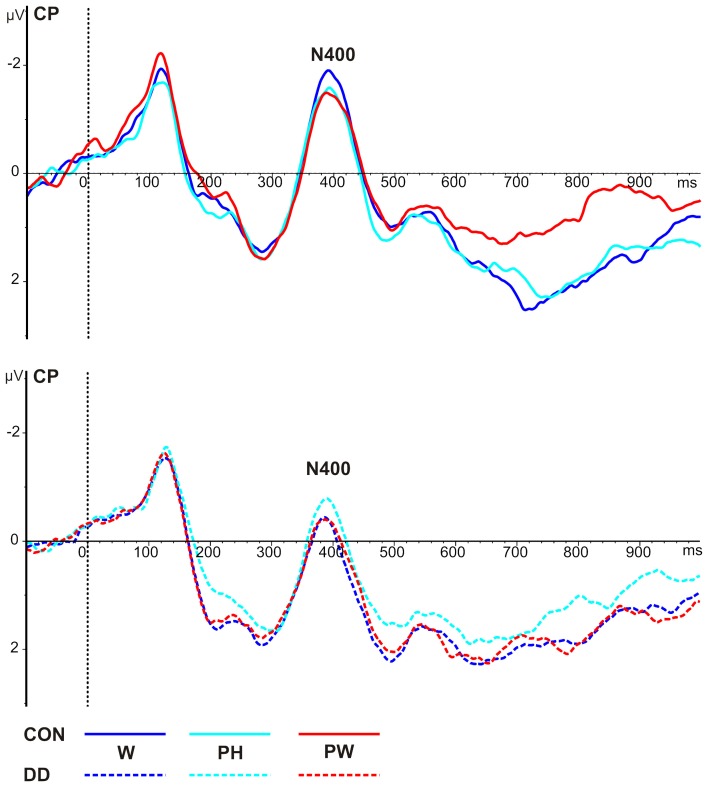
**Illustration of the averages across centro-parietal (CP) electrodes included in the ROI of the N400 for control children (CON) and children with DD (DD).** W, words; PH, pseudohomophones; PW, pseudowords. Negativity is depicted upwards.

***Mean peak amplitudes.*** N400 mean peak amplitudes were more negative in the control group compared to the group with DD (main effect *group, F*_(1, 79)_ = 5.34, *p* = 0.023, η_*p*_^2^ = 0.06; see Table 4 and Figure 5). N400 mean peak amplitudes were comparable high for all *conditions*, *F*_(2, 158)_ = 0.28, *p* = 0.75, and no significant interaction between *group* and *condition*, *F*_(2, 158)_ = 0.68, *p* = 0.51, could be observed.

**Table 4:**
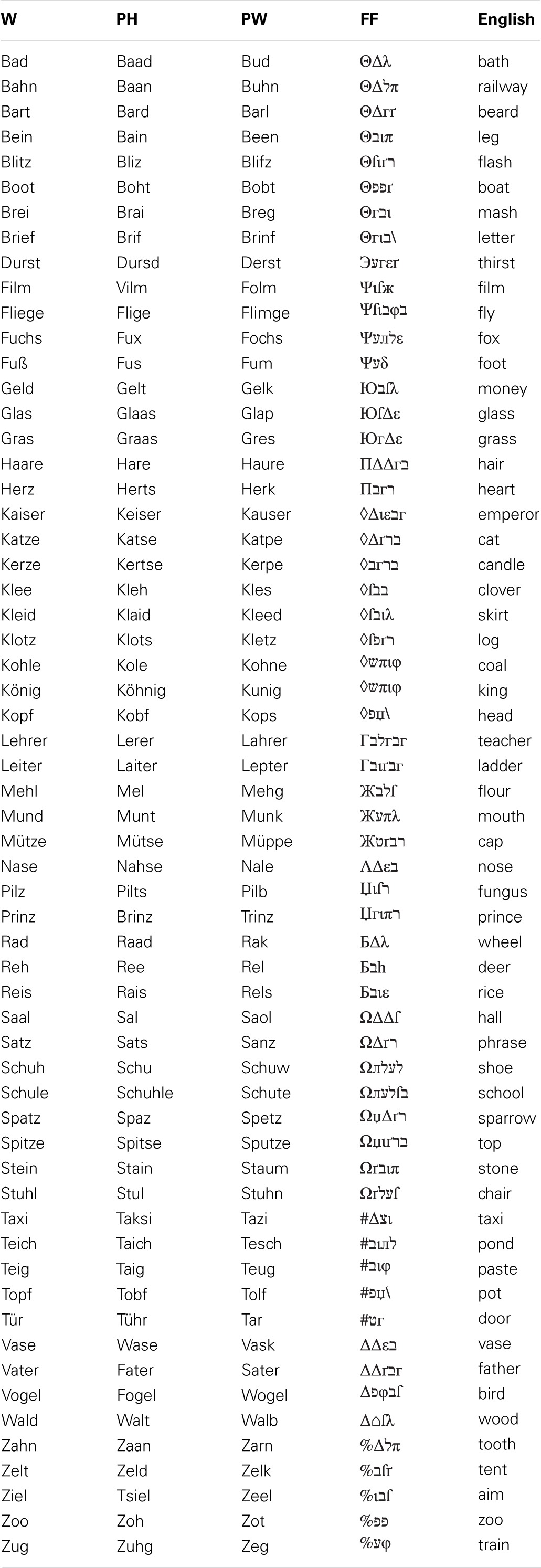
**N400 mean peak amplitudes (μV) and latencies (ms)**.

W, words; PH, pseudohomophones; PW, pseudowords; CON, control children; DD, children with DD.

***Peak latencies.*** N400 peak latencies did not differ between *groups*, *F*_(1, 79)_ = 1.49, *p* = 0.23, and *conditions*, *F*_(2, 158)_ = 1.53, *p* = 0.22, and no significant interaction between *group* and *condition*, *F*_(2, 158)_ = 2.76, *p* = 0.07, could be observed (see Table 4 and Figure 5).

#### LPC

***Area under the curve.*** A main effect *condition*, *F*_(2, 158)_ = 4.41, *p* = 0.014, η_*p*_^2^ = 0.05, occurred. Furthermore, a significant twofold interaction between the factors *condition* and *group* was observed, *F*_(2, 158)_ = 4.05, *p* = 0.019, η_*p*_^2^ = 0.05. Independent *post-hoc t*-tests revealed no significant differences between the groups (W, *t*_(79)_ = 1.32, *p* = 0.19; PH, *t*_(79)_ = 1.69, *p* = 0.09; PW, *t*_(79)_ = −1.14, *p* = 0.26).

**Figure 6:**
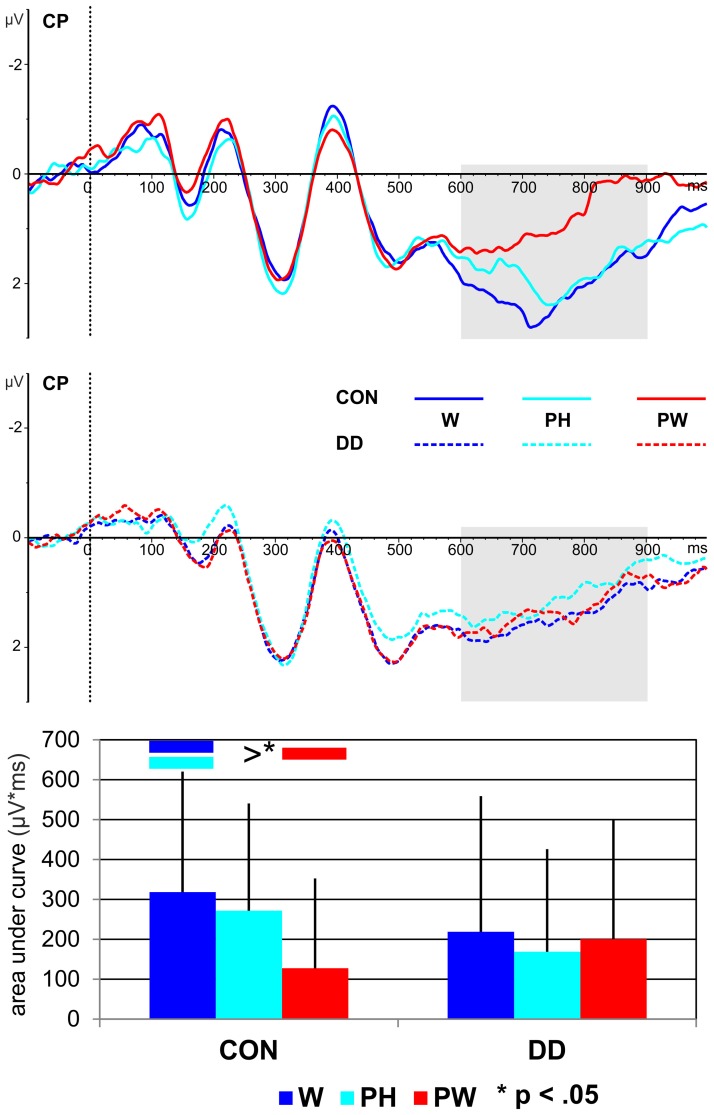
**Illustration of the averages across centro-parietal (CP) electrodes included in the ROI of the LPC for control children (CON) and children with DD (DD) and illustration of the mean area under the curve.** W, words; PH, pseudohomophones; PW, pseudowords. The time window selected for the LPC is highlighted in gray (600–900 ms). Negativity is depicted upwards. Error bars illustrate standard deviations.

As can be seen in Figure 6 only in the control group more activation for both W and PH compared to PW was found (dependent *post-hoc t*-tests: W vs. PW, *t*_(28)_ = 3.57, *p* = 0.001, *d* = 0.66; PH vs. PW, *t*_(28)_ = 2.63, *p* = 0.014, *d* = 0.49). The activation for W and PH was comparable high in control children (dependent *post-hoc t*-test: W vs. PH, *t*_(28)_ = 0.91, *p* = 0.37). Conditions did not differ in the group with DD (dependent *post-hoc t-tests:* W vs. PH, *t*_(51)_ = 1.25, *p* = 0.22; W vs. PW, *t*_(51)_ = 0.37, *p* = 0.71; PH vs. PW, *t*_(51)_ = −0.78, *p* = 0.44; see Figure 6).

### Behavioral data of the PLD—task

#### Reaction times

**Figure 7:**
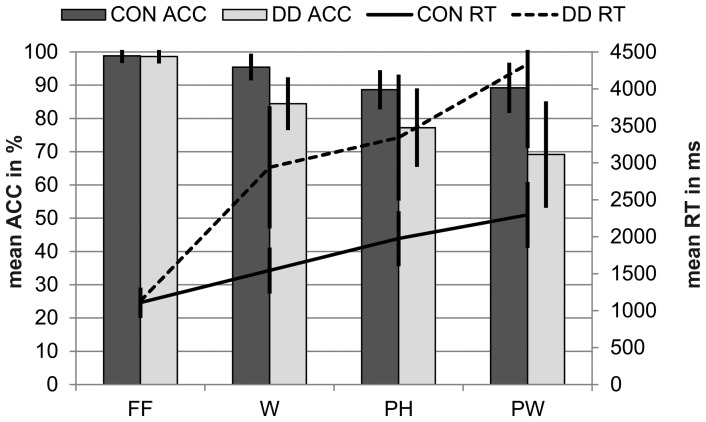
**Behavioral results for the PLD—task for control children (CON) and children with DD (DD).** ACC, accuracy; RT, reaction time; FF, false fonts; W, words; PH, pseudohomophones; PW, pseudowords. Error bars illustrate standard deviations.

Performance on the PLD—task revealed a reaction time difference between *conditions*, *F*_(1.77, 139.63)_ = 323.85, *p* < 0.001, η_*p*_^2^ = 0.80, and *groups*, *F*_(1, 79)_ = 80.84, *p* < 0.001, η_*p*_^2^ = 0.51. Furthermore, a significant twofold interaction between the factors *condition* and *group* occurred, *F*_(1.77, 139.63)_ = 68.38, *p* < 0.001, η_*p*_^2^ = 0.46. Control children had smaller reaction times to W, *t*_(71.81)_ = −10.90, *p* < 0.001, *d* = 2.68, PH, *t*_(75.70)_ = −9.99, *p* < 0.001, *d* = 2.40 and PW, *t*_(72.86) = −11.46_, *p* < 0.001, *d* = 2.80, compared to children with DD. There was no difference between groups regarding reaction times to FF, *t*_(79)_ = −0.49, *p* = 0.63 (see Figure 7).

*Post-hoc t*-tests within each group revealed the same pattern of reaction times for both groups. Both control children and children with DD had longer reaction times for all linguistic stimuli compared to FF (CON: W vs. FF, *t*_(28)_ = 9.45, *p* < 0.001, *d* = 1.75; PH vs. FF, *t*_(28)_ = 16.31, *d* = 3.03, *p* < 0.001; PW vs. FF, *t*_(28)_ = 15.83, *p* < 0.001, *d* = 2.94; DD: W vs. FF, *t*_(51)_ = 16.78, *p* < 0.001, *d* = 2.33; PH vs. FF, *t*_(51)_ = 20.05, *p* < 0.001, *d* = 2.78; PW vs. FF, *t*_(51)_ = 21.24, *p* < 0.001, *d* = 2.95). In both groups reaction times were shorter for W compared to PH (CON, *t*_(28)_ = −12.70, *p* < 0.001, *d* = 2.36; DD, *t*_(51)_ = −7.81, *p* < 0.001, *d* = 1.08) and PW (CON, *t*_(28)_ = −15.12, *p* < 0.001, *d* = 2.81; DD, *t*_(51)_ = −14.24, *p* < 0.001, *d* = 1.97). And both groups responded slower to PW compared to PH (CON, *t*_(28)_ = 7.60, *p* < 0.001, *d* = 1.41; DD, *t*_(51)_ = 12.54, *p* < 0.001, *d* = 1.74).

#### Accuracy

Performance on the PLD—task revealed an accuracy difference between *conditions*, *F*_(2.23, 175.83)_ = 96.92, *p* < 0.001, η_*p*_^2^ = 0.55, and *groups*, *F*_(1, 79)_ = 50.83, *p* < 0.001, η_*p*_^2^ = 0.39. Furthermore, a significant twofold interaction between the factors *condition* and *group* occurred, *F*_(2.23, 175.83)_ = 21.67, *p* < 0.001, η_*p*_^2^ = 0.22. Independent *post-hoc t*-tests revealed that control children's performance was significantly better in all linguistic conditions compared to the performance of the group with DD (W, *t*_(78.42)_ = 8.32, *p* < 0.001, *d* = 1.96; PH, *t*_(78.27)_ = 5.80, *p* < 0.001, *d* = 1.37; PW, *t*_(77.39)_ = 7.65, *p* < 0.001, *d* = 1.81; see Figure 7). No group differences were found for the FF-condition, *t*_(79)_ = 0.36, *p* = 0.72.

Dependent *post-hoc t*-tests within each group revealed that control children gave more correct answers to FF compared to all linguistic stimuli (W vs. FF, *t*_(28)_ = −3.92, *p* = 0.001, *d* = 0.73; PH vs. FF, *t*_(28)_ = −8.55, *p* < 0.001, *d* = 1.59; PW vs. FF, *t*_(28)_ = −7.71, *p* < 0.001, *d* = 1.43). Furthermore, control children's accuracy was higher to W compared to PH and PW (W vs. PH, *t*_(28)_ = 9.09, *p* < 0.001, *d* = 1.69; W vs. PW, *t*_(28)_ = 4.54, *p* < 0.001, *d* = 0.84) and accuracy rates did not differ between PH and PW for control children, *t*_(28)_ = −0.41, *p* = 0.69. Similarly to the control children, dependent *post-hoc t*-tests revealed that children with DD gave more correct answers to FF compared to all linguistic stimuli (W vs. FF, *t*_(51)_ = −13.08, *p* < 0.001, *d* = 2.43; PH vs. FF, *t*_(51)_ = −13.16, *p* < 0.001, *d* = 2.44; PW vs. FF, *t*_(51)_ = −13.87, *p* < 0.001, *d* = 2.58). Furthermore, accuracy rates were higher to W compared to PH and PW (W vs. PH, *t*_(51)_ = 5.59, *p* < 0.001, *d* = 0.78; W vs. PW, *t*_(51)_ = 7.95, *p* < 0.001, *d* = 1.10) and higher to PH compared to PW, *t*_(51)_ = 3.99, *p* < 0.001, *d* = 0.55, in the group with DD.

### Correlational results

When interpreting the correlation results, please note that the mean area under the curve for the N170 difference waves and the N400 mean peak amplitudes have negative values. No correlation was found between the mean area under the curve for the N170 difference waves and the performance in reading and spelling. N400 mean peak amplitudes were correlated with spelling (*r* = −0.25, *p* = 0.025), indicating that better spelling was related to enhanced N400 mean peak amplitudes. Furthermore, a smaller LPC mean area under the curve for PH was correlated with better spelling (*r* = −0.22, *p* = 0.048).

## Discussion

The present study was designed to investigate the single processing steps underlying PLDs in order to provide a temporal model of reading processes in normal developing children and to further clarify which processing steps are degraded in children with DD during reading. Therefore, we decided to employ a PLD—task in children with and without DD while recording their neurophysiological activity via EEG. Children were presented with W, PH, PW and FF and had to decide via button press whether the presented stimuli sounded like a real German word or not. In the following sections we will relate our ERP findings to single processing steps suggested by dual route models of reading, thus providing a temporal model of reading processes for children. Furthermore, deficits related to single processing steps in DD will be discussed and clinical implication for intervention derived from our findings will be offered.

### Temporal model of reading processes in normal developing children and deficits in DD

Dual route models of reading (Coltheart et al., 1993, 2001) suggest that reading proceeds in a hierarchical manner. After the completion of visual and orthographic processing steps phonology of a letter string can be accessed in different ways depending on the orthographic familiarity of the letter string. Familiar known words are read first by accessing the orthographic representations in the orthographic lexicon and then by retrieving the corresponding phonological representations from the phonological lexicon. Unfamiliar word forms, such as pseudohomophones and pseudowords or familiar words for which the reader does not possess an entry in the orthographic lexicon are read by applying grapheme-phoneme correspondence rules in order to access the phonological representation.

Although reading models assume different processing steps they do not provide information about the time course of single processing steps. However, knowledge about when single processing steps occur is important, especially in order to achieve a better understanding which processing steps during reading are degraded in subjects with DD and how these deficits might lead to the reading speed deficit, which is suggested to be the main criterion to diagnose DD in transparent orthographies.

#### N170 indexes orthographic processing and is deficient in DD

At about 220 ms the child's brain differentiates between orthographic (W, PH, PW) and non-orthographic control stimuli (FF) as indicated by higher mean peak amplitudes for orthographic stimuli compared to FF. This effect of print sensitivity can be allocated to the first processing step of reading models, namely the visual-orthographic processing step. In accordance with previous studies the N170 was distributed equally over the left and the right occipito-temporal scalp (see Figure 3) and delayed for about 50 ms (Maurer et al., 2006; Spironelli and Angrilli, 2009; Kast et al., 2010; Hasko et al., 2012) compared to the adults left lateralized N170 (Bentin et al., 1999; Maurer et al., 2005a,b), indicating that this first processing step is not yet fully automated in children. According to the phonological mapping hypothesis (McCandliss and Noble, 2003; Maurer et al., 2010) processing of written language becomes left lateralized with increasing reading experience during development because phonological processes, which mediate grapheme-phoneme conversion, are typically left lateralized. This hypothesis is supported by a longitudinal study from Maurer and colleagues (2006), who were able to show that print sensitivity develops with reading instruction. While the N170 amplitudes were comparably high for words and symbol strings in preschool children, children at the end of second grade showed an effect of print sensitivity and N170 was distributed equally across hemispheres for words (Maurer et al., 2005b, 2006). Furthermore, Maurer and colleagues observed a clear shift of N170 amplitudes to the left hemisphere in adults (Maurer et al., 2005b, 2006). Thus, our results further indicate that the development of print sensitivity is not completed in third graders and suggest that the underlying system for fast visual word recognition is not yet entirely automated.

In contrast to a previous study by Maurer et al. (2007) children with DD in the present study, similar to control children, had higher mean peak amplitudes to orthographic compared to non-orthographic control stimuli. Children had a mean age of eight years in both studies. However, in the present study children were at the beginning of grade three, whereas children in the Maurer et al. (2007) study attended grade two, thus emphasizing the influence of increasing reading experience on the N170 as well as the plasticity of the N170 in DD. Although children with DD in the present study showed an effect of print sensitivity in the mean peak amplitudes the degree of print sensitivity was reduced as indicated by a significantly diminished mean area under the curve for the difference waves compared to children without DD (see Figure 4A). This finding also corresponds to fMRI studies investigating the PLD—task in subjects with DD and showing a lack of print sensitivity in the VWFA (Wimmer et al., 2010), next to a general hemodynamic hypoactivation of the VWFA (van der Mark et al., 2009; Wimmer et al., 2010), which is thought to generate the N170 (Allison et al., 1994; Tarkiainen et al., 1999; Salmelin et al., 2000). Overall, reduced VWFA activity was repeatedly reported for tasks requiring visual processing of words in subjects with DD (e.g., Démonet et al., 2004; Shaywitz and Shaywitz, 2008; Richlan et al., 2009).

Whereas the N170 was distributed equally across hemispheres, the degree of print sensitivity was more pronounced over the left hemisphere in both groups as indicated by greater mean areas under the curve over the left hemisphere compared to the right hemisphere (see Figure 4A). The greater left hemispheric activation of the difference waves is probably due to slightly higher activations for FF in the right hemisphere (see Table 3; amplitude means are at about 1 μV higher in the right hemisphere), which is in line with previous studies showing a tendency toward right hemispheric processing for non-orthographic material (e.g., Bentin et al., 1999; Maurer et al., 2008). In order to compute the difference waves the activation to FF was subtracted from the orthographic material, thus resulting in a greater difference between orthographic material (W; PH; PW) and FF in the left hemisphere compared to the right hemisphere.

Thus far, fMRI studies examining the PLD—task reported an orthographic familiarity effect. Orthographic familiarity refers to a higher activation for unfamiliar (PH and PW) letter strings, compared to familiar letter strings (W) in the VWFA in normal developing subjects. This effect was absent in subjects with DD (van der Mark et al., 2009; Wimmer et al., 2010). Furthermore, some electrophysiological studies also reported an orthographic familiarity effect for the N170, i.e., lower N170 amplitudes for words with higher orthographic familiarity (Compton et al., 1991; McCandliss et al., 1997; Sereno et al., 1998; Hauk and Pulvermüller, 2004). These findings suggest that in this point in time the orthographic lexicon is accessed at least in adult readers. However, other studies did not replicate amplitude differences between words, pseudowords or consonant strings in children (Maurer et al., 2005b; Kast et al., 2010) and adults (Nobre et al., 1994; Salmelin et al., 1996; Bentin et al., 1999; Cornelissen et al., 2003). The orthographic familiarity effect seems to also depend on task demands as indexed by a study of Bentin et al. (1999), who found differences between consonant strings and words during explicit lexical and semantic tasks but not during implicit reading. Although the children in the present study had to explicitly read the word in order to resolve the task they did not show an orthographic familiarity effect. N170 mean peak amplitudes were comparable high for W, PH and PW (see Table 3 and Figure 3). Furthermore, the mean area under the curve for the difference waves, measuring the degree of orthographic familiarity were negligible not only for children with DD but also for control children (see Figure 4B).

There are two possible explanations for the lack of orthographic familiarity in control children in the present study. Firstly, Barber and Kutas (2007) proposed that the sensitivity to orthographic familiarity might be dependent on the stimulus material included in the experiment. Studies including only orthographic material, which varied in orthographic familiarity, found an orthographic familiarity effect in the N170 time window, whereas studies additionally investigating non-orthographic material reported an influence of orthographic familiarity in a later time window, namely the N400. Barber and Kutas (2007) suggested that by presenting only orthographic material the human brain might prepare to process the presented stimuli as orthographic, thus accelerating reading processes. Accordingly, the lack of orthographic familiarity in the N170 in the present study might be explained by the investigation of both orthographic material (W, PH, PW) and FF. However, as we investigated 8-year-old children, it might be more likely that the lack of orthographic familiarity could be ascribed to the lower level of reading experience. This assumption is supported by a study of Kast and colleagues (2010), who explored a visual lexical decision task in 10-year-old children. Children were presented with words and pseudowords and had to decide whether the presented stimulus was a word or not. Although Kast et al. (2010) only investigated orthographic material they did not find an orthographic familiarity effect in the N170 concluding that this might be the result of lower reading experience in children and a less established reading system (Kast et al., 2010).

To summarize, the control children's brain differentiates orthographic familiar (W, PH, PW) from non-orthographic control stimuli (FF) at about 220 ms. However, there was no effect of orthographic familiarity in this early time window suggesting that reading processes at this point in time might be comparable for orthographic familiar and unfamiliar word forms in young children and further proposing that the orthographic lexicon has not yet been accessed. With respect to children with DD the degree of print sensitivity was reduced and points to deficits in this early stage of reading processes and at this age.

#### N400 indexes comparable reading processes for W, PH and pw and points to deficits in DD

According to hierarchical reading models the next processing step comprises the access to the orthographic lexicon in case of familiar word forms (W) and the applying of grapheme-phoneme correspondence rules in case of unfamiliar word forms (PH; PW) respectively in order to access phonology in a last step of reading process. Dual route models of reading (Coltheart et al., 1993, 2001) suggest that the search for an orthographic representation in the orthographic lexicon and the appliance of grapheme-phoneme correspondence rules occur in a parallel manner. In adults it has been found that N400 amplitudes were smaller to orthographic familiar word forms compared to unfamiliar word forms (e.g., Braun et al., 2006; Briesemeister et al., 2009). These results suggest that less effort was needed in order to find a fitting orthographic representation for familiar words in the orthographic lexicon, whereas the search was prolonged and grapheme-phoneme correspondence rules had to be applied in case of unfamiliar word forms resulting in enhanced N400 amplitudes.

In line with previous studies the N400 was distributed over centro-parietal electrodes in the present study (Deacon et al., 2004; for review see Lau et al., 2008; Kutas and Federmeier, 2011). In contrast to the N400 orthographic familiarity effect reported in adults N400 mean peak amplitudes were comparable high for W, PH and PW in the present study. Our findings are in accordance with results of previous studies investigating children (e.g., Coch et al., 2002; Coch and Holcomb, 2003). For example, in the study of Coch and Holcomb (2003) 7-year-old children were required to read word lists consisting of stimuli which varied with respect to orthographic familiarity (i.e., real words with differing degree of difficulty for 7-year-old children and nonpronounceable letter strings) and had to respond via button press whenever an animal name was presented. The authors did not report a modulation of the N400 by orthographic familiarity (Coch and Holcomb, 2003). These results together with the findings of the present study suggest that children rely on comparable reading processes for all letter strings independent of orthographic familiarity. Furthermore, as we did not find an effect of phonological familiarity in the time window of the N400, i.e., amplitude differences between phonological familiar (W; PH) and unfamiliar word material (PW), the present study contradicts the assumption that the N400 might reflect post-lexical processing at least in young children (Kutas and Hillyard, 1980; Brandeis et al., 1994; Schulz et al., 2008; for review see Lau et al., 2008; Kutas and Federmeier, 2011).

In children with DD the N400 was nearly absent in the present study (see Table 4 and Figure 5). Reduced N400 activation in subjects with DD has been reported previously (Ackerman et al., 1994; Brandeis et al., 1994; Johannes et al., 1995; Rüsseler et al., 2007; Schulz et al., 2008). The assumption that the N400 might index the searching process for an orthographic representation in the orthographic lexicon and the appliance of grapheme-phoneme correspondence rules is further strengthened by the partial correlation results. Better spelling performance was correlated to higher N400 mean peak amplitudes irrespective of being diagnosed with DD or not. A prerequisite for correct spelling is both knowledge of grapheme-phoneme correspondence rules and knowledge of orthographic rules (Klicpera et al., 2007). Thus, the correlation between correct spelling and N400 mean peak amplitudes suggests that children at this point in time might be engaged with applying grapheme-phoneme correspondence rules or the searching process for an orthographic representation in the orthographic lexicon. Diminished N400 amplitudes in children with DD point to deficits in these processes. This conclusion is in line with both the phonological (Snowling, 2001; Ramus et al., 2003; Vellutino et al., 2004) and the orthographic core deficit (e.g., Bergmann and Wimmer, 2008; Bekebrede et al., 2009; van der Mark et al., 2009) reported for children with DD in transparent orthographies. According to the phonological deficit hypothesis it is assumed that subjects with DD have difficulties in manipulating and applying grapheme-phoneme correspondence rules. According to the orthographic core deficit an impaired or delayed access to available orthographic representations or poorer and less specified orthographic representations (Bergmann and Wimmer, 2008; Bekebrede et al., 2009; van der Mark et al., 2009; Marinus and de Jong, 2010) are suggested.

To summarize, control children's N400 mean peak amplitudes suggest that children at the age of eight years rely on comparable reading processes for W, PH and PW, as there was no effect of orthographic familiarity in the N400 time window. With respect to children with DD, N400 amplitudes were significantly reduced indicating less specified orthographic representations or impairments in accessing the orthographic lexicon and in applying grapheme-phoneme correspondence rules.

#### LPC indexes phonological lexical access in control children and is degraded in DD

According to hierarchical reading models the last processing step includes the access to the phonological lexicon. In the present study the phonological lexicon was accessed between 600 and 900 ms after stimulus onset in control children as indicated by a phonological familiarity effect for the LPC. That is, the mean area under the curve of the LPC did not differ between W and PH, which share the same phonological representation, but was significantly reduced for PW, which do not have an entry in the phonological lexicon. Interestingly, a small correlation between the LPC mean area under the curve for PH and spelling was found, indicating that independent of group better spelling is correlated to smaller activation for PH. The correlation suggests that orthography has an influence even in this late time window. When inspecting the grand average of the LPC (see Figure 6) the activation for PH sharing the same phonological representations with W, but violating the orthographic representation seems to lie between the activation for W and PW, although this does not reach significance. It is possible that children at this stage of reading acquisition are not aware of all orthographic violations posed by PH and might accept PH to be orthographically correct. Thus, it might be speculated that children with more reading and spelling experience might show a decreasing activation pattern from W over PH to PW. In line with previous studies the LPC was distributed over left centro-parietal electrodes (Friedman and Johnson, 2000; Hepworth et al., 2001; Finnigan et al., 2002; Rüsseler et al., 2003; Schulte-Körne et al., 2004; Yonelinas et al., 2005; van Strien et al., 2009; Balass et al., 2010; for review see: Rugg and Curran, 2007). The allocation of the LPC to the left hemisphere is not surprising, as left hemispheric activation has been repeatedly reported for tasks requiring phonological processing (e.g., Price et al., 1997; Rumsey et al., 1997; Shaywitz et al., 2002; Shaywitz and Shaywitz, 2008).

In children with DD the LPC did not differentiate between phonological familiar and phonological unfamiliar word forms (see Figure 6). Because previous studies investigated word recognition tasks a direct comparison with our results is not possible. Nevertheless, deficient activation of LPC has also been reported in adolescents (Schulte-Körne et al., 2004) and adults (Rüsseler et al., 2003) with DD in word recognition tasks. For example, in the experiment by Schulte-Körne et al. (2004) participants with and without DD were required to learn pseudoword lists in a first phase and had to indicate in a second phase whether the presented pseudoword was a learned pseudoword or not. The LPC was found to be higher to learned compared to new pseudowords in control children only. This was interpreted as reflecting a specific word recognition memory deficit in DD. In the present study, however we did not examine word recognition and the phonological familiarity on the LPC in control children was interpreted as indicating access to the phonological lexicon. Therefore, the absence of a modulation of the LPC by phonological familiarity might indicate an impaired access to phonological representations or an underspecification of phonological representations (Ramus and Szenkovits, 2008).

To summarize, control children's LPC suggests that at this point in time the phonological lexicon is accessed. With respect to children with DD the lack of phonological familiarity on the LPC indicates an impaired access to phonological representations or an underspecification of phonological representations.

### Behavioral data mirrors the core deficits of young children with DD

Overall our results on the behavioral level mirror the main characteristics of DD in transparent orthographies, namely a rather high reading accuracy, which is accompanied by severe deficits in reading speed. Children with DD in the present study displayed rather high accuracy rates (between 70 and 85%), however they were substantially delayed in their reaction times for all lexical conditions compared to control children. There is evidence that the reading speed deficit observed in subjects with DD in transparent orthographies can be traced back to a persistent reliance on the non-lexical route (e.g. De Luca et al., 2002; Zoccolotti et al., 1999, 2005). However, it has been shown that the reading speed deficit in DD can be ascribed to both non-lexical and lexical route reading (Bergmann and Wimmer, 2008). Children (Moll and Landerl, 2009) and adolescents (Bergmann and Wimmer, 2008) with DD do indeed engage in visual whole word processing and read via the lexical route for orthographically known words, but their reading speed is impaired. Thus, the prolonged reaction times in the present study and the response pattern, which was similar to control subjects (FF < W < PH < PW) suggest that subjects with DD might rely on comparable reading processes as control children at least for some items. Overall the behavioral results in the present study replicate findings of former studies (Bergmann and Wimmer, 2008; van der Mark et al., 2009, 2011; Wimmer et al., 2010). Compared to the children examined in the study of van der Mark et al. (2009, 2011) reaction times were longer in both control children and children with DD in the present study. This is probably due to age differences. Children in the study of van der Mark et al. (2009, 2011) were three years older and had more reading experience. Proportionally, however, the speed impairment of subjects with DD compared to control subjects remained stable across both studies. This is in line with longitudinal studies, showing that the gap between skilled and less skilled readers in reading performance still remains over time although both high and poor performers develop in word reading (e.g., Klicpera et al., 1993; Shaywitz et al., 1999).

### Limitations of the study

One limitation of the present study is that the behavioral data does not match to the ERP data. Whereas the reaction time results suggest that children might use orthographic representations for reading orthographic familiar word material (W) and might rely on grapheme-phoneme correspondence rules for orthographic unfamiliar word material (PH; PW) we were not able to detect different reading processes depending on orthographic familiarity in the ERP data. Neither the N170, nor the N400 showed an orthographic familiarity effect in form of lower amplitudes for orthographic familiar compared to orthographic unfamiliar word forms. In contrast, fMRI studies did find support for engaging both routes in children, adolescents and adults (Kronbichler et al., 2007; Bruno et al., 2008; van der Mark et al., 2009; Wimmer et al., 2010).

We would like to offer three explanations for the discrepancy observed between our behavioral and ERP data. Although van der Mark et al. (2009) did report an orthographic familiarity effect in children, the children in the present study were three years younger and less experienced readers, suggesting that one possible explanation for the lack of orthographic familiarity in the ERP might be the younger age of the children investigated in the present study. It has been proposed that the orthographic familiarity effect is the result of reading experience (Reicher, 1969). It might be that the effect of orthographic familiarity is only partly developed in 8-year-old children as it has been observed on the behavioral level but not in the ERP data. Another possible explanation might be that children differ with respect to their reading development, as indicated by great variance of the ERP measures, even though they were very similar with respect to reading performance, IQ and age on the behavioral level, thus masking an effect of orthographic familiarity. Enlarging the sample size might have reduced the variance observed in the ERP data and may have led to an effect of orthographic familiarity. A third explanation for the absence of an orthographic familiarity effect in the ERP data might be that children rely on comparable reading processes for W, PH, and PW, however after having accessed the phonological representation they need more time to decide whether the presented word exists or not. This is supported by the long reaction times, especially for PW. Whereas the LPC indicates that the phonological lexicon has been accessed between 600–900 ms after stimulus onset, most children responded to PW more than one second later, suggesting that they might have been insecure whether the presented stimuli was a real word or not. Longitudinal studies are necessary in order to better understand the discrepancies between the behavioral and ERP findings and in order to clarify at which age and reading level an orthographic familiarity effect can be also observed in the ERP data.

## Conclusion

In the present study we attempt to provide a temporal model of reading processes in normal developing children by relating our ERP findings to single processing steps suggested by dual route models of reading in order to clarify which processing steps are degraded in children with DD during reading. ERPs provide evidence for deficient processes from the very first processing stage until the last processing stage. To summarize, a reduced mean area under the curve for the word material-false font contrasts in the time window of the N170 suggested a reduced degree of print sensitivity. Furthermore, diminished N400 amplitudes pointed to less specified orthographic representations or to deficits in accessing the orthographic lexicon and in applying grapheme-phoneme correspondence rules. And lastly, the lack of phonological familiarity on the LPC indicated an impaired access to phonological representations or an underspecification of phonological representations. These deficits are in line with the orthographic and phonological core deficit reported for subjects with DD in transparent orthographies. The results of our study suggest that effective treatment should include both orthographic and phonological training. In general more longitudinal studies and studies investigating adults utilizing the same task and stimuli are needed to clarify how the observed processing steps and their time course change during reading development and how they differ from mature reading processes, which in turn has major implications on reading instructions in school and in therapeutic settings for children with DD.

### Conflict of interest statement

The authors declare that the research was conducted in the absence of any commercial or financial relationships that could be construed as a potential conflict of interest.

## Appendix

Stimuli of the phonological lexical decision task; W, words; PH, pseudohomophones; PW, pseudowords; FF, false fonts and English translation.
